# Addressing biomedical data challenges and opportunities to inform a large-scale data lifecycle for enhanced data sharing, interoperability, analysis, and collaboration across stakeholders

**DOI:** 10.1038/s41598-025-90453-x

**Published:** 2025-02-21

**Authors:** Vivek Sriram, Ashley Mae Conard, Ilyana Rosenberg, Dokyoon Kim, T. Scott Saponas, Amanda K. Hall

**Affiliations:** 1 Department of Biostatistics, Epidemiology and Informatics, Perelman School of Medicine, University of Pennsylvania, Philadelphia, PA 19104 USA; 2 Genomics and Computational Biology Graduate Group, Perelman School of Medicine, University of Pennsylvania, Philadelphia, PA 19104 USA; 3 Health Futures, Microsoft Research, Microsoft Building 99, 14820 NE 36Th Street, Redmond, Washington 98052 USA; 4 Institute for Biomedical Informatics, University of Pennsylvania, Philadelphia, PA 19104 USA; 5 Department of Biomedical Informatics and Medical Education, University of Washington School of Medicine, Seattle, WA 98195 USA

**Keywords:** Biomedical discovery, Multiomics, Precision medicine, Data interoperability, Research data lifecycle, Personalized medicine, Computational platforms and environments, Data mining, Data processing, Standards

## Abstract

Biomedical discovery is fraught with challenges stemming from diverse data types and siloed analysis. In this study, we explored common biomedical data tasks and pain points that could be addressed to elevate data quality, enhance sharing, streamline analysis, and foster collaboration across stakeholders. We recruited fifteen professionals from various biomedical roles and industries to participate in sixty-minute semi-structured interviews, which involved an assessment of their challenges, needs, and tasks as well as a brainstorm exercise to validate each professional’s research process. We applied a qualitative analysis of individual interviews using an inductive-deductive thematic coding approach for emerging themes. We identified a common set of challenges related to procuring and validating data, applying new analysis techniques and navigating varied computational environments, distributing results effectively and reproducibly, and managing the flow of data across phases of the data lifecycle. Our findings emphasize the importance of secure data sharing and facilities for collaboration throughout the discovery process. Our identified pain points provide researchers with an opportunity to align workstreams and enhance research data lifecycles to conduct biomedical discovery. We conclude our study with a summary of key actionable recommendations to tackle multiomic data challenges across the stages and phases of biomedical discovery.

## Introduction

Achieving tailored medical treatment for every patient is a significant goal of biomedical research. Given the data diversity and various stakeholders involved, fulfilling this vision necessitates a shared process for biomedical discovery. *Biomedical discovery* involves the investigation of disease etiology and the elucidation of underlying mechanisms of biological processes. *Precision medicine* aims to achieve a more accurate and precise version of medicine that uses large-scale, multi-modal data to characterize the underlying mechanisms of disease onset across cohorts of patients and improve outcomes in clinical settings. The ultimate goal of precision medicine is to transform patient care through individualized disease prediction, prevention, treatment, and therapeutics^1,2^.

The currency for both precision medicine and biomedical discovery has always been data. Precision medicine begins with the integration of *multiomics datasets*, data that correspond to different levels of biological structure^3^, for the purpose of gaining a comprehensive understanding of human health. These insights can assist healthcare professionals in personalizing patients’ diagnoses and treatment plans^4^.

The advent of *big data*, the exponential increase in variety and quantity of data that are collected, has significantly disrupted the field of biomedical discovery, leading to a rapid increase in the pace of innovation^5^. In only the past few years, big data facilitated the complete sequencing of the human genome^6^, pioneered chimeric antigen receptor (CAR) T-cell therapy for cancer^7^, and contributed to the development of novel vaccines during the COVID-19 pandemic^8^.

Despite the many advances made across the field of biomedical discovery, a lack of data interoperability and an absence of a unified standard across biomedical data types has left the ultimate promise of precision medicine unfulfilled. Therapeutic medicine has remained largely unchanged over the past twenty years, with minimal benefits to public health and ever-expanding research and development costs and researchers across the pipeline of biomedical discovery unable to align on a common process for accelerated research^2^. The majority of initiatives to improve the pace of biomedical research focus on advanced tooling and do not address challenges in data flow and collaboration across stakeholders^9,10^.

There is an opportunity to explore a unified process for biomedical research that facilitates enhanced data sharing, interoperability, analysis, and collaboration. Although contextual nuances vary extensively, we can identify a consistent set of data “jobs to be done” across subdisciplines of biomedical discovery. Across all subdisciplines of precision medicine research, researchers are handling large-scale, complex, high-dimensional data that include a variety of heterogeneous formats^11^. These data are typically isolated within their respective institutions, hindering reproducibility and preventing efforts to generate diverse, longitudinal, comprehensive patient cohorts.

A variety of stakeholders are involved in biomedical discovery and precision medicine research, including healthcare systems, clinical laboratories, technology companies, academia, and government^12^. The promise of precision medicine and the development of accurate biomedical digital twins rely on the ability of these stakeholders to collaborate with one another and accurately link diverse, high-quality data across ‘omic subtypes. Without a shared workstream to process and validate data collected from multiple studies, the output of biomedical data will not be as usable to new knowledge discovery.

Each biomedical subdiscipline assumes that its work differs from the rest. However, if we could identify similarities across data modalities and converge on a unified process for biomedical discovery research, then we could drastically reduce the time required to develop an individualized understanding of disease. Only through participation from stakeholders across basic sciences, translational research, clinical, and public health can we hope to reach a unified process to deliver population-level health benefits.

Many biomedical discovery frameworks have been published that aim to unify research workstreams (Table 1, Supplemental Table 1)^13–31^. However, each of these frameworks addresses only a specific research context related to tooling needs and data analysis. They also presume quality and integrity of the data. One notable example of a framework that has successfully reduced the time spent on research development is the drug discovery process – however, this process is specific to drug development and does not include other therapeutic or AI precision medicine discoveries. None of the other frameworks capture the full scale of biomedical discovery across data modalities and stakeholder roles while also considering the scope of data interoperability and integrity (Table 1, Supplemental Table 1).

**Table 1:** A summary of some identified frameworks in the healthcare space.

Paper	Data types considered	Personas considered	Scope of framework	Summary
A framework for big data technology in health and healthcare	Healthcare data (healthcare provider data, EMRs, insurance company/payer data, patient data, wearables)	N/A	Clinical research	Summarize options for clinical data sources, big data storage and analysis systems, and translational opportunities for clinical data in a 4-step process
A framework for the use of genomics data at the EPA	Genomic data	N/A	Human non-clinical research and disease diagnosis	Set of guidelines to be considered when working with genetic data
A Harmonized Data Quality Assessment Terminology and Framework for the Secondary Use of Electronic Health Record Data	Clinical data from EHRs	N/A	Clinical research	Multiple studies evaluating data quality in clinical research were harmonized to construct a unified set of requirements
An Integrated Data Management Framework for Drug Discovery – From Data Capturing to Decision Support	Chemical data related to drug discovery and development	N/A	Drug Discovery	Drug discovery informatics platform that allows for management of multiple reagents compounds, and assays
Argonaut: A Web Platform for Collaborative Multiomic Data Visualization and Exploration	Multiomics data	N/A	Data visualization and analysis for multiomics research	Secure, web-based sharing of data analysis and visualization for multiomics data
Assuring the Machine Learning Lifecycle: Desiderata, Methods, and Challenges	Data agnostic	N/A	Machine Learning Analysis	Defined a 4-step process / iterative loop for the lifecycle of machine learning analysis

Broadening our perspective beyond frameworks focused on biomedical research, multiple models have been published that focus on the research data life cycle at large, including Carlson 2014, Ball 2012, Cox and Tam 2018, Sinaeepourfard et al. 2016, and Möller 2013^32–36^. While these more general data lifecycle models do not include context specific to biomedical data, they serve as effective baselines that can be adapted to reflect broader biomedical discovery across data modalities and stakeholder types through the inclusion of facets unique to the challenges of the field. In other words, we can adapt such frameworks to provide guidelines for how participants should standardize each step of analysis to expedite different stages of biomedical research. Regulatory bodies such as the FDA could use an adapted biomedical research data lifecycle to clarify their expectations regarding biomedical discovery, facilitating a simplified submission process for research groups and a more thorough cycle of data validation and verification. Indeed, using such an updated framework could help foster data interoperability across the landscape of biomedical investigation through its definition of a unified procedure for research.

### Study objectives

Data quality and interoperability are imperative in biomedical research. This need motivated our study to better understand the overarching process of biomedical discovery research across stakeholders and biomedical data types. Thus, our objectives were to (1) identify and define the processes and tasks performed by biomedical researchers, (2) evaluate researchers’ needs and challenges related to data, data management, and collaboration, and (3) assess the analytical tools and workflows that researchers leverage to conduct their work.

## Materials and methods

We conducted fifteen sixty-minute semi-structured interviews with individuals placed throughout the scope of biomedical discovery, including computational biologists, research scientists, data curators, data stewards, and data generators. The first part of each interview focused on the participant’s background, research objective, general tasks and jobs-to-be-done, data and tooling needs, and current challenges. The second part of each interview focused on a brainstorming exercise. We present details on participant recruitment, informed consent process, data collection, and analysis methods below.

### Ethics statement and participant recruitment

We conducted our study with fifteen professionals who work in biomedical discovery research in the United States (US). Our study criteria consisted of participants of age range 18 to 100, who work in biomedical discovery in the US, and speak English. Our study (protocol ID 10415 was reviewed and approved by Microsoft Research Institutional Review Board (IRB). Written informed consent was obtained from each participant prior to the start of the interviews. All interviews were conducted in accordance with relevant guidelines and regulations.

Participants were enrolled through a research recruitment company that recruits for studies across the US. Participants were recruited through a combination of methods including active outreach and internal study panel contact databases. A detailed participant screener was applied, and pre-approval was performed by the research team. Pre-approved and interested participants who met study eligibility criteria were informed about the purpose of the study and provided a copy of the informed consent. Interested participants who provided written informed consent to the research recruitment company were then scheduled for an interview. Prior to the start of each interview session, participants were asked if they had any questions related to the study and confirmed they had read and signed the informed consent. Participants were compensated $175 USD for their time via a gift card distributed through the research recruitment company.

### Data collection

**Fig. 1:**
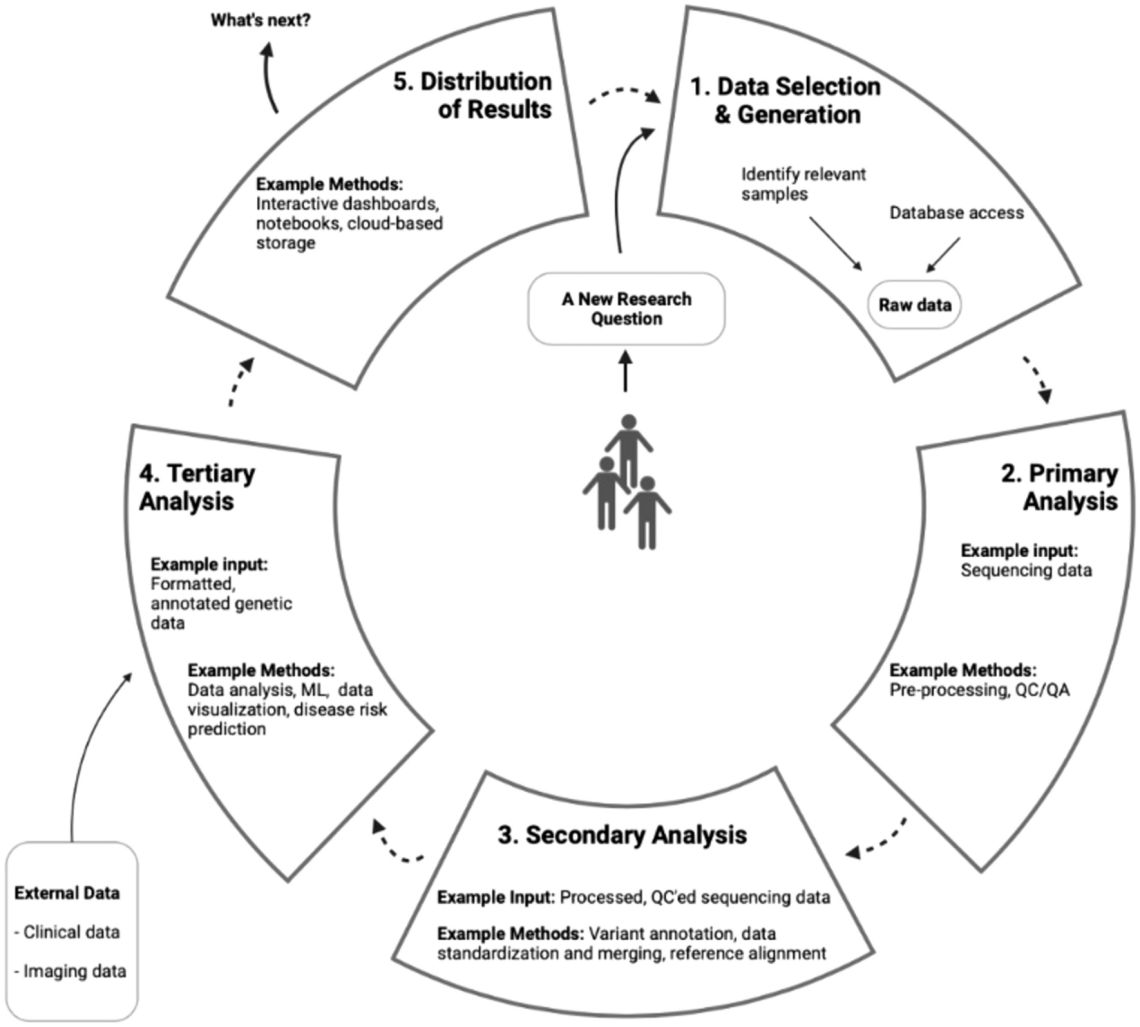
Baseline visualization for brainstorming exercise. Created with Biorender.com.

**Fig. 2:**
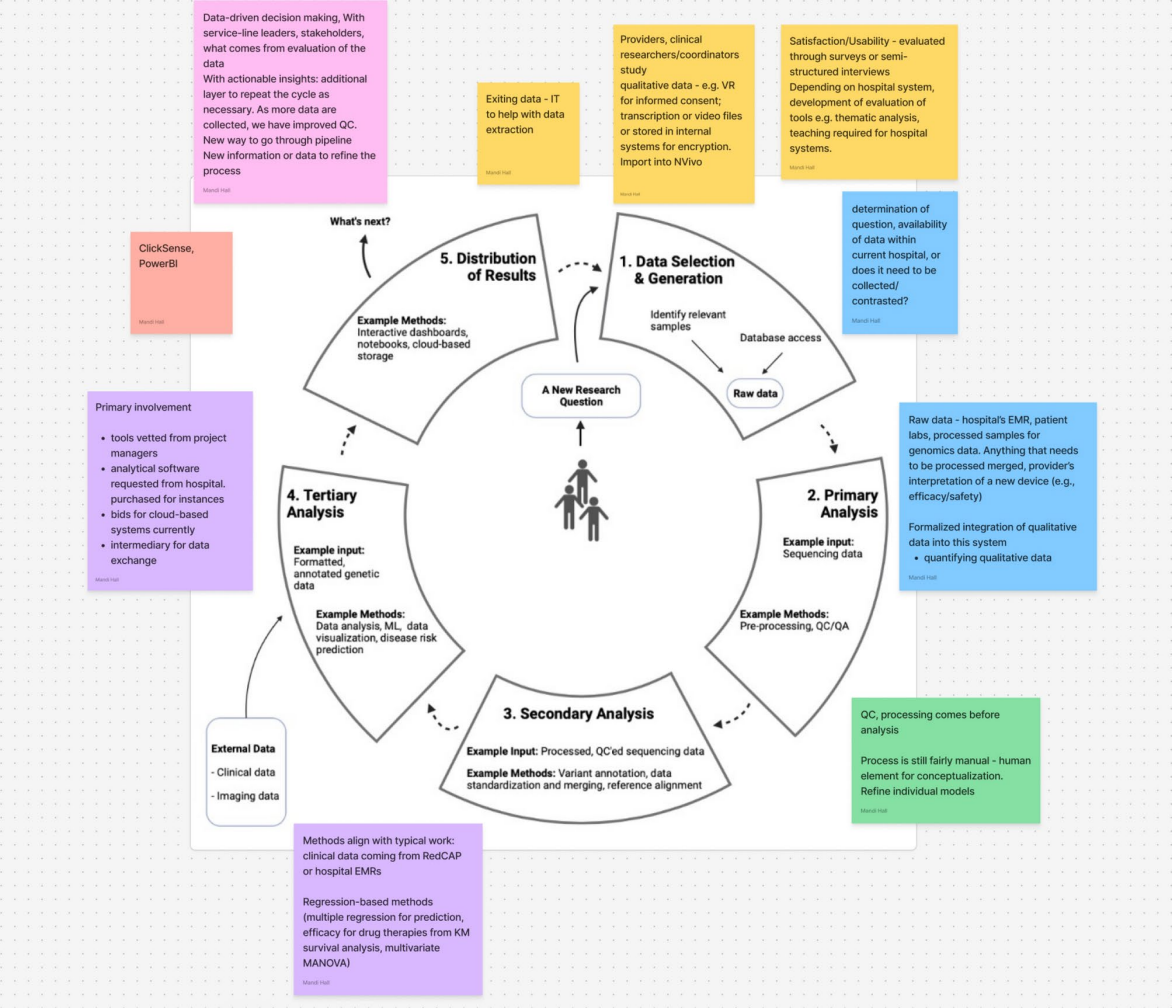
Example of the note-taking process during the brainstorm exercise. Created with Biorender.com.

In the first half of each interview, participants were asked questions related to their professional roles, the type of work they conduct, the research problems they are trying to solve, the data and tools they use, their challenges and needs, and their day-to-day research tasks. In the second half of the interview, the research team displayed a research diagram (Fig. 1) on their screen and asked questions related to how similar or different the diagram flow was to the participants’ research processes, where in the diagram flow they would position their day-to-day roles, and what information was amiss as well as what suggestions they had for how to accurately represent each stage of their research process. Figma (https://www.figma.com) was used for the virtual whiteboard brainstorm portion of the interview and sticky notes were used to capture participants’ feedback in real-time to allow them to clarify and validate their research process. Figure 2 depicts an example of the notetaking process. We created our research diagram (Fig. 1) as a brainstorm tool to elicit feedback from participants during the interviews to validate their research process^37,38^. At the end of each interview, participants were asked general quantitative demographic questions.

### Data analysis

All interviews were conducted via Microsoft Teams video conference platform from July to August of 2022 by the first author (VS) and audio-recorded with participants’ informed consent. Microsoft Teams auto transcription was used and then each interview transcript was verified and corrected for accuracy later via the recordings by authors VS and AKH. The first and last authors met periodically to discuss interviews and identify emerging themes. We applied a combination of inductive and deductive thematic coding approaches to the qualitative data^39^. Initial themes consisted of ‘data collaboration,’ ‘data quality,’ and ‘phases of analysis.’ As the interviews progressed, we iterated over the data to produce higher-level themes, such as ‘data extraction’ and ‘access’, ‘clinical trial data platforms’, ‘analysis processes’, and ‘data hand-offs’.

## Results

### Participant demographics

All participants lived in the US, worked in biomedical discovery research, and worked with a range of nonclinical, clinical, imaging, and genomics data. The age range of participants were 18–24 (1), 25–34 (8), 35–44 (4), and 45–54 (2). Their work experience ranged from 1–5 years (5), 5–10 years (4), and more than 10 years (6). Our study included 5 females and 10 males. Most participants identified as Caucasian/European descent (9), followed by South Asian (3), East Asian (1), African Descent (1), and other/Mixed Ancestry (1). Participants worked in a variety of industry and academic settings ranging in size from self-employed freelance positions to companies with over 20,000 employees, with about half coming from pharmaceutical or biotechnology operations and the other half from academic medical centers, healthcare organizations, or hospitals.

### Participant expertise

The participants in this study included good laboratory practice (GLP) / benchwork scientists, good clinical practice (GCP) researchers, sequencing core personnel, dry lab scientists, and clinicians. Each individual had different understandings and uses of biomedical data based on their expertise and practice settings. We summarize these varied interpretations in Table 2.

**Table 2:** Expertise and practice settings for study participants.

Interviewee Category	Number of representative individuals from interviews	Expertise	Practice setting(s)	Examples of interpretations of biomedical data
GLP / Benchwork scientists	2	Lab technicians for early-stage and nonclinical research	Non-profit research center Academic medical center	Nonclinical, assay-based data
GCP researchers /curators	5	Regulatory guidance vendor Cohort builders / clinical research assistants Data engineer – health outcomes IT administrator	Pharmaceutical companies Academic medical centers Radiology company Third-party data vendor	Clinical trial outcomes Patient health criteria MRI scans
Sequencing core personnel	1	Bioinformatics analyst	Immunology lab at a research university	Preprocessed genomic sequencing files
Dry lab scientists	5	Third-party statisticians and biotechnicians consulting for clinical trial data analysis	Biotechnology and pharmaceutical companies	Unstructured clinical data (i.e. medical notes)
Clinicians	2	Clinician conducting research Pharmacist facilitating treatment evaluation	Research institution Medical center	Patient responses to new care equipment and treatments

As Table 2 suggests, different sectors of stakeholders in the biomedical discovery process have vastly different definitions of biomedical data depending on the roles that they play. Nevertheless, while these distinct subsets and uses of biomedical data all require separate normalization processes and data structures, the flow of data from non-clinical discovery to downstream precision medicine research necessitates a unification of data processes and enhanced collaboration among all personas.

### Qualitative findings

The most common research motivations that participants discussed during interviews were the development of new domain-specific insights to (a) identify cohorts for clinical trials, (b) accelerate drug development, (c) bring therapeutics to patients, (d) facilitate FDA regulatory approval, (e) simplify patient diagnosis, and (f) discover positive changes that could be implemented in clinical settings for improved patient health outcomes.

Participants described a variety of data types with which they worked (Table 3), including protein abundances from model organisms, structured and free-text clinical data, genomic single-cell and whole genome sequencing data, and post-clinical data, such as drug performance and marketing metrics.

**Table 3:** Data Types used by Participants.

Data Types Used
• ELISA / FISH / Flow cytometry data from model organisms and patient tissue samples
• Clinical data from patient electronic health records o Lab measurements, vital readings, biomarker/metabolite measures, imaging/radiology data, qualitative measurements
• Genomic data o Single-cell RNA-sequencing (scRNA-seq) o Whole Genome DNA-sequencing (WGS)
• Post-clinical data o Drug performance data o Drug marketing data

Analysis tools were highly context-dependent (Supplemental Table 2) – participants used IBM SPSS, REDCap, and Microsoft Excel for intuitive computation, ImageJ and Prism for image analysis, GATK for primary and secondary genomic data, Python (including pandas, NumPy, SciPy packages), R (including Bioconductor, as well as ggplot2 and other tidyverse libraries), SQL, and SAS for general data needs, Nextflow and Cromwell for pipelining and workflow development, and Anaconda and Docker for versioning of software environments.

### Challenges related to biomedical discovery

Based upon the interviews from our qualitative study, we identified the following pain points that typically hinder the biomedical discovery process.

#### Challenge 1. Identifying and procuring the appropriate data for a given research question

A primary focus across participant interviews was navigating the balance between identifying and extracting the appropriate data for a given research question**.** Both sufficient financial resources and an adequate amount of time are needed to either generate the required data or to procure it from an external source. Particularly in experimental lab (“wet lab”) environments, paper-based data collection can be a tedious manual process for much of data generation, leading to increased risk of downstream quality issues when transferring data into computational environments. Furthermore, complications can arise in terms of coordination and collaboration among stakeholders and research planners to identify the most suitable data for the research question at hand.

#### Challenge 2. Curating and validating procured data for downstream analysis

Ensuring the integrity and quality of procured data was another major concern across interviews. Pain points highlighted during the data curation process include lag time during data curation, particularly when processing unstructured data, a lack of consistency in the requirements for data quality control across organizations and biomedical subfields, an absence of effective, privacy-compliant data sharing methods, and tedious manual data processing when transferring data across systems to collaborators and stakeholders, particularly with respect to clinical research.

#### Challenge 3. Learning how to apply new analysis methods to validated data and navigating inconsistent computational environments

Participants coming from more traditional biological and medical backgrounds described facing significant learning curves when attempting to design and apply computational analysis workflows for the first time. Participants also mentioned a lack of standardized processes for version control of code and data. Interviewees working specifically with large-scale ‘omics data described how the scale of their data can make analysis and debugging in local environments infeasible. Participants working in computational biology research described how they needed to use both Python and R environments for their analysis work, and that continually transitioning back and forth between the two platforms was often an ordeal. Ultimately, both the variety of coding environments and software and the lack of effective, user-friendly methods for multiomics data integration hamper research participants’ ability to conduct reproducible analysis, adding to the time required for data analysis in the biomedical discovery process.

#### Challenge 4. Distributing data-driven findings effectively and reproducibly

The hope of interviewees in the distribution of the results of their data-driven analysis was that the results generated by data-driven discovery could be used to advance broader knowledge in the field. Key challenges with respect to the distribution of results included meeting regulatory requirements for data output, ensuring reproducibility of generated workflows and results, validating biological interpretation of results, and appropriately conveying the significance and meaning of conclusions drawn to public audiences.

#### Challenge 5. Managing the flow of data across phases of the data lifecycle

The numerous methods described by participants for storing (Supplemental Table 3), sharing (Supplemental Table 4), and managing access (Supplemental Table 5) highlight the significance of data flow from generation and procurement to curation and validation to analysis and discovery. Key pain points identified with respect to the data handoffs that occur among stakeholders included a lack of unity among data management and sharing systems, prohibitive data storage costs, difficulties ensuring data privacy and security, inconsistent regulatory requirements, learning curves for new data storage systems, a lack of standardization in version control expectations for code and data, and bottlenecking and latency due to the need for coordination among multiple stakeholders.

Ultimately, across the challenges identified for biomedical discovery, participant interviews all echoed a single message: **the significance of collaboration and trust surrounding the flow of data**. Each exchange of data involved multiple professional stakeholders, including data generators, research scientists, data curators, third-party vendors, bioinformaticians, computational biologists, biologists, and clinicians. Insight and interpretation are continually needed from all stakeholders involved to ensure the accuracy and integrity of the data.

### Recommendations

Based upon our data analysis findings, we developed a list of seven key actionable recommendations for organizations looking to enhance their ability to conduct biomedical discovery research.

#### Recommendation 1. Create a user-friendly platform for bench-side data collection in biological research

A transition from manual to electronic data collection in biologic discovery could increase efficiency, improve trust in the data collection and data analysis process for bench-side scientists, and improve interplay between wet and dry lab research.

#### Recommendation 2. Establish a unified system for reproducible biomedical research

A unified system for data analysis could allow for consistent, sharable workflows and lead to a lower barrier to entry for computational analysis. An example of a group implementing such a system is the single-cell community, which consistently makes use of the Seurat and Monocle packages for its research. Furthermore, having such a system could help stakeholders keep track of data input and research progress throughout the biomedical discovery pipeline.

#### Recommendation 3. Develop a simplified workflow for debugging and integration from notebooks into workflows to handle the large scale of ‘omics data

This workflow could include the option to version control markdown documents and notebooks, as well as a graphical user interface to facilitate debugging in the cloud.

#### Recommendation 4. Study the third-party data management vendor networks for drug development

Currently, the robustness of the IT infrastructure for a project can vary extensively depending on the organization in charge – larger companies tend to have stronger, cloud-based infrastructures for data storage and administration. More data mean more complications in terms of data processing, data transfer, and analysis, and in such situations, multiple experts from a variety of fields are required to manage the data. Third-party data management vendors are highly useful in managing these data access issues as well as facilitating regulatory proceedings for pharmaceutical companies. A better understanding of the systematized data exchange that occurs across these could vastly expedite biomedical discovery.

#### Recommendation 5. Introduce improved, user-friendly tooling for data processing and ingestion

Multiple opportunities lie in the ability to use methods such as generative AI for data processing^40,41^. Integrating natural language processing and machine learning with the latest transformer or large language models could help reduce data loss through the processing of unstructured free-form text. Furthermore, tools that incorporate generative AI could reduce the learning curve for more complicated data processing techniques by providing direct feedback on data processing workflows for users jumping into computational analysis for the first time. Intuitive, user-friendly tools would help democratize access to data and simplify the ability to ingest them for downstream data analysis.

#### Recommendation 6. Improve the process of communication between clinical trial managers and clinicians

The wide variation in the data sharing systems that are used across pharmaceutical companies and third-party vendors results in a tremendous burden on clinical trial facilitators to ensure the ongoing viability of the trial – multiple data management portals may be required for a project depending on the type of data being used. Clinicians and other healthcare providers are also often not able to directly see the impact of the work they help facilitate. We could reduce the turn-around time for biomedical discovery in the clinical space through the development of easy-to-use co-working platforms that facilitating effective collaboration and communication between clinical trial managers and clinicians.

#### Recommendation 7. Develop tooling and platforms to facilitate quicker data access and more efficient, secure data sharing

The creation of secure, democratized data platforms that permit rapid, secure data sharing both within and beyond an organization would drastically help mitigate the existing challenges in data flow. Such tooling would need to include cost-efficient data storage and options for secure communication and data transfer between internal and external parties.

## Discussion

Data integrity and interoperability are essential to improve our ability to achieve precision medicine. However, most of the research conducted today is fixated on the development of new tools and methods for analysis. This myopic focus ignores the gaps in biomedical experimentation that lead to failings of data interoperability. To identify these omissions, our work aimed to explore the biomedical discovery process across professional stakeholder roles, research goals, and data subtypes. Our qualitative study provided insights into the data journey across stakeholders involved in biomedical discovery, and based on the identified data challenges, we proposed a set of seven actionable recommendations for those interested in addressing open challenges in the discipline.

Our study findings confirm many of the data challenges found in the literature with respect to biomedical discovery research, such as concerns related to (1) secure data storage, warehousing, withdrawal, access, and sharing, (2) quality control and curation of unstructured data, (3) processing and multiomics integration for large-scale, heterogeneous data, (4) reproducibility and version control, (5) coordination and collaboration among stakeholders, and (6) regulatory standards and collaborative data partnerships. Finally, we identified the importance of data integrity in hand-offs between the stages of biomedical research, and noted how data integrity is often assumed by data professionals who do not collect and curate their own data due to the isolation of their work from other steps in the research process ecosystem. Based on such considerations, we present an overview of a research data lifecycle that reflects the diverse data types, stages, and stakeholders involved in the biomedical discovery process derived from our study findings (Table 4).

**Table 4:** An overview of biomedical discovery framed within a research data lifecycle, derived from the results of our interviews.

Phase	**STAGE 1:** DATA PLAN	**DATA HANDOFF:** RAW NON-CURATED DATA	**STAGE 2:** DATA CURATION AND PREPROCESSING	**DATA HANDOFF:** QUALITY-CONTROLLED PROCESSED DATA	**STAGE 3:** DATA ANALYSIS	**DATA HANDOFF:** ROBUST FINDINGS	**STAGE 4:** DATA-DRIVEN SOLUTION / DISCOVERY
**LEVEL A** **NON-HUMAN** **Stakeholder Segments** - Academic Medical Centers / Research Centers - Pharma / Biotech - Vendor Networks	- If data are available, get a vendor to send them over - If data are unavailable, pay a data generator to create the data - Data collection and data standardization typically occur concurrently	- Handoff from data generator to data curator or computational scientist Requires FDA compliance if proceeding to human studies	- Further curation and collection of metadata (e.g. handling batch effects)	- Handoff from a data generator to computational scientist	- Statistical analysis from a computational scientist	- Insight / interpretation from computational scientist and other stakeholders - Shared through presentations, shared drives, visualizations, publications	- Communication with collaborators - Publication of manuscript and/or data / workflows to advance the field
**LEVEL B** **HUMAN, NON-CLINICAL** **Stakeholder Segments** - Academic Medical Centers / Research Centers - Pharma / Biotech - Vendor Networks	- Collaboration with a wet lab or large-scale data consortium - If data are available, procure tissue samples or sequencing information - Otherwise, perform sequencing / data extraction	- Handoff occurs from the lab technician or an external vendor to the computational scientist	- Performed by a computational scientist - For ‘omics data, follow best practices for QC and annotation – primary (reference alignment) and secondary analysis (variant calling) - Short-tailed process	- Data evaluation continued by computational scientist or biologist - Back-and-forth communication circle	- Context-dependent data analysis - Involves statistical/ML modeling as well as biological interpretation - Incorporation of public knowledge - Long-tailed process	- Insight / interpretation from computational scientists, biologists, and other stakeholders - Shared through presentations, shared drives, visualizations, publications, markdown notebooks, or file sharing systems	- Communication with collaborators - Publication of manuscript and/or data / workflows to advance the field
**LEVEL C** **HUMAN, CLINICAL** **Stakeholder Segments** - Hospital Systems / Academic Medical Centers - Pharma / Biotech - Vendor Networks	- Raw data collection from patients - Mediation from third-party data management vendors (CDMs) - Long-tailed process	- Data go from a health system to a data management vendor to the pharmaceutical sponsor	- Cleaning / reformatting data for use in analysis, performed by a data scientist or data curator - Oversight from a data management entity is often required - Long-tailed process	- Curated data are handed to either a third-party computational scientist or an internal data curator or computational scientist	- Evaluation of efficacy of treatment	- Insight / interpretation from clinicians, pharmaceutical representatives, data analysts, and other stakeholders - Shared through presentations, publications, dashboards, or vendor-specific portals that help manage data	- Enact change in medical centers and distribute results to the broader community - Meet regulatory expectations - Cycle of validation and verification

Based on the above research data lifecycle, we include an additional itemization of biomedical data tasks by stages of research, including non-clinical and clinical discovery (Table 5).

**Table 5:** Overview of data tasks by stage of biomedical research.

Stage	Main Tasks	Subtasks
*Forming a new research question*		
Stage 1: Data Plan Collaborative stage with multiple stakeholders that work together to determine data needs and how best to answer research questions; Mainly asking—Is there data available or do we need to collect data to answer our research questions?	1. Data collection (private data)	a. Clinical data (samples collected from patients)
		b. Non-clinical data (lab results from tissue samples from model organisms)
	2. Data extraction (public and private data, and data access)	a. Individual-level sequencing data
		b. Clinical data (Electronic Health Records, Claims, Payer data, Imaging)
		c. Large-scale biobanks, social determinants of health
*Data Hand-off: Raw non-curated data*		
Stage 2: Data Curation and Preprocessing	1. Quality control (generated data)	a. Manual process of checking for errors, experts in the loop to validate output, handle batch effects, etc
	2. Data standardization (generated and controlled data)	a. Label metadata
		b. Correct file formats (fixed naming conventions)
		c. Curate data
*Data Handoff: Quality-controlled, processed data*		
Stage 3: Data Analysis	1. Re-formatting data	a. Merge multiple datasets and types of data
	2. Apply methods	a. Identify the right tools and execute
		b. Hypothesis testing
		c. Ensure reproducibility
	3. Investigation	a. Validate or invalidate research questions
		b. Develop new insights
*Data Handoff: Robust findings*		
Stage 4: Data-Driven Discovery Stakeholders come together to review results and discuss next steps	1. Review results	a. Contribute to common/collective knowledge
		b. Determine if new data or more data are needed
		c. Return to Phase 3
	2. Identify next steps	a. Develop new research question(s)
		b. Submit to regulatory agencies for clinical trial approval
		c. Change polices based on findings
*Output: AI Models, Therapeutics, or New Discoveries*		

The results of our study emphasize the need for secure collaboration and data analysis, with a focus on reducing data handoff miscommunication, early-stage data extraction errors, metadata errors, and reformatting errors during analysis to meet compliance and regulatory standards. Our study was limited by the logistics of recruiting participants, as it had to be conducted virtually. As a result, further work is needed to validate the identified obstacles and data jobs to be done with additional participants. Furthermore, our proposed recommendations and process must be explored in real-world settings. Another future direction of this work involves additional investigation of the third-party data management and contract research organization vendor networks to gain a full grasp of the data flow and hand-offs that take place in larger-scale biomedical discovery projects.

## Conclusion

In this study, we explored key challenges and data jobs to be done from the perspective of a variety of biomedical researchers. Based on the results of our interviews, we identified a set of common pain points and challenges faced by researchers across the biomedical discovery data lifecycle. We proposed a set of recommendations for improved data collaboration, integrity, and interoperability for knowledge discovery, including cloud-based computational infrastructures for centralized data warehousing and withdrawal, improved debugging workflows for the analysis of large-scale heterogeneous data, new methods for the ingestion of unstructured data, and the establishment of vendor networks to facilitate data management and the fulfilment of regulatory requirements. Such developments will be crucial to ensure the accuracy and reproducibility of biomedical models when considering transitions toward production-level applications in healthcare and the life sciences. Furthermore, we highlighted an example biomedical discovery process incorporating findings from our interviews that demonstrates how stakeholders across various sectors of biomedical analysis could converge on a common workflow to enhance data sharing and foster collaboration.

More research is needed to validate if our proposed recommendations could enhance existing data lifecycle frameworks to improve large-scale multiomics data integrity, interoperability, analysis, and collaboration challenges. Through their application, we hope to see a shift in biomedical discovery research practices, bringing us closer to realizing individualized therapeutics for all patients and fulfilling the promise of precision medicine.

## Supplementary Information

Supplementary Information.

## Data Availability

Aggregated findings from this qualitative study can be shared upon reasonable request to the corresponding author.
