# Supplementary material for: Addressing biomedical data challenges and opportunities to inform a large-scale data lifecycle for enhanced data sharing, interoperability, analysis, and collaboration across stakeholders
Source: Sci Rep. 2025 Feb 21;15:6291. doi: 10.1038/s41598-025-90453-x (PMC11845626; doi:10.1038/s41598-025-90453-x)
Supplement: Supplementary file 1 — Supplementary Information. [file 41598_2025_90453_MOESM1_ESM.docx]

**SUPPLEMENTAL DATA**

Supplementary Table 1. A list of all identified frameworks in the healthcare space

| **Paper** | **Data types considered** | **Personas considered** | **Scope of framework** | **Summary** |
| --- | --- | --- | --- | --- |
| **A framework for big data technology in health and healthcare** | Healthcare data (healthcare provider data, EMRs, insurance company/payer data, patient data, wearables) | N/A | Clinical research | Summarize options for clinical data sources, big data storage and analysis systems, and translational opportunities for clinical data in a 4-step process |
| **A framework for the use of genomics data at the EPA** | Genomic data | N/A | Human non-clinical research and disease diagnosis | Set of guidelines to be considered when working with genetic data |
| **A Harmonized Data Quality Assessment Terminology and Framework for the Secondary Use of Electronic Health Record Data** | Clinical data from EHRs | N/A | Clinical research | Multiple studies evaluating data quality in clinical research were harmonized to construct a unified set of requirements |
| **An Integrated Data Management Framework for Drug Discovery – From Data Capturing to Decision Support** | Chemical data related to drug discovery and development | N/A | Drug Discovery | Drug discovery informatics platform that allows for management of multiple reagents compounds, and assays |
| **Argonaut: A Web Platform for Collaborative Multiomic Data Visualization and Exploration** | Multiomics data | N/A | Data visualization and analysis for multiomics research | Secure, web-based sharing of data analysis and visualization for multiomics data |
| **Assuring the Machine Learning Lifecycle: Desiderata, Methods, and Challenges** | Data agnostic | N/A | Machine Learning Analysis | Defined a 4-step process / iterative loop for the lifecycle of machine learning analysis |
| **Best practice data life cycle approaches for the life sciences** | Genetic sequencing and annotations, metabolite/proteomic profiles | Research scientists | Life sciences / Biomedical / Biosciences / Bioinformatics Research | Best-practice lifecycle for life sciences / biomedical data, highlighting data reusability |
| **Best practice framework for Patient and Public Involvement (PPI) in collaborative data analysis of qualitative mental health research: methodology development and refinement** | Qualitative mental health data | Patient and Public Involvement (PPI) Researchers | Mental Health research | Multi-stage framework for qualitative research in the mental health space |
| **Building Highly-Optimized, Low-Latency Pipelines for Genomic Data Analysis** | Genomic Sequencing data | N/A | Aligned/annotated genetic read analysis for association and causality | Multi-step pipeline for QC and analysis of genomic sequencing data |
| **Clinical data quality: a data life cycle perspective** | Clinical data | N/A | Clinical research, clinical trial. Recruitment, phenotype-driven rare/genetic disease research, large-scale observational studies | Multi-stage data life cycle for clinical data quality, use, and reuse |
| **Cloud-based Healthcare data management Framework** | Clinical data (from unstructured patient data to EHRs) from healthcare organizations | N/A | Cloud-based management and analysis of healthcare data | Cloud Infrastructure for the ingestion, storage, and processing of clinical data |
| **Defining and Developing a Generic Framework for Monitoring Data Quality in Clinical Research** | Clinical data | N/A | Evaluate quality of data in clinical research using this framework | A “Fit-for-use” data quality monitoring framework, presented as a nested concentric network, can facilitate increased efficiency in clinical data quality monitoring |
| **Development of a Genomic Data Flow Framework: Results of a Survey Administered to NIH-NHGRI IGNITE and eMERGE Consortia Participants** | Genomic data | Patients, Genomic researchers, Clinicians | Interpretation of genetic profiles in clinical settings | A mapped exploration of data processing and analysis for clinical interpretation of patient genetic information |
| **Ethics of Using and Sharing Clinical Imaging Data for Artificial Intelligence: A Proposed Framework** | Patient data | Patients, Researchers, Clinicians, Administrators, Payers, Purchasers, Industry | Secondary use of data (research) requires researchers to act as ethical data stewards | Set of ethical considerations as. Medical imaging data are used for research |
| **Gathering and Learning from Relevant Clinical Data: A New Framework** | Clinical data | N/A | Standardize clinical practice | Theory-built framework called the Standardized Clinical Assessment and Management Plan (SCAMP) |
| **Impact of a five-dimensional framework on R&D productivity at AstraZeneca** | Metabolite/pharmacological data | N/A | Pharmaceutical research / drug discovery | 5R framework (Right target, tissue, safety, patient, and commercial potential) can accelerate the process of drug discovery for big pharma |
| **Preparing Medical Imaging Data for Machine Learning** | Radiology / Patient Imaging Data | AI researchers | Making medical image data available for ML/AI research into improving radiology diagnosis | Defined steps for process of medical image data handling |
| **Security model for Big Healthcare Data Lifecycle** | Clinical data (from unstructured patient data to EHRs) from healthcare organizations | N/A | Identify weak security points in the lifecycle of healthcare data | Multi-stage data lifecycle going from data collection to knowledge creation for healthcare research using big data |
| **State of the Field in Multi-Omics Research: From Computational Needs to Data Mining and Sharing** | Omics data – genomics, epigenomics, transcriptomics, proteomics, metagenomics, etc. | N/A | Non-clinical research – defining best practices for FAIR sharing | A flow diagram defining best steps for data analysis, sharing, and reproducibility in a FAIR ecosystem |
| **Translational Research 2.0: a framework for accelerating collaborative discovery** | Life sciences / biomedical data | Medical / Life science researchers, care delivery organizations | Represent the overarching ecosystem of biomedical and life sciences research | A depiction of the translational sciences ecosystem |

Supplementary Table 2. Tools Used by Participants

| **Tools Used** |
| --- |
| - User-friendly data analysis - IBM SPSS, REDCap, Epic, Excel/Macros |
| - Image analysis - ImageJ, Prism |
| - Evaluating data quality - AWS Deequ |
| - scRNA-seq - Seurat, Monocle, ScanPy |
| - Primary and secondary genomic data analysis - GATK: best practice workflow |
| - Tertiary / General Data Analysis - Python (pandas, numpy, scipy, Jupyter notebooks, VSCode) - R (Bioconductor, ggplot, tidyverse, RMarkdown, RStudio) - SQL - SAS |
| - Genomics-specific analysis - Google Genomics Pipelines API on GCP - Glow genomics toolkit on AWS Spark |
| - Pipelining and workflow development - Nextflow, Cromwell |
| - Heavy data analysis - RAPIDS framework, Vaex.io, IPyWidgets, CUDA Python (CuPy) |
| - Versioning - Anaconda, Docker |

Supplementary Table 3. Participant Methods for storing data

| **Methods for Storing Data** |
| --- |
| - Microsoft Excel, Box cloud storage, SharePoint, OneDrive |
| - Watson LIMS |
| - On-premises clusters |
| - Epic/Epic CareConnect |
| - REDCap |
| - Benchling |
| - Amazon Web Services (AWS) RedShift/S3 |
| - Microsoft SQL Server |
| - Snowflake |
| - Google Cloud Platform (GCP) |
| - Third-party data vendors / EDC vendor-based portals - Medidata RAVE, Inform, IBM Clinical, Open Clinica, Triad - FlatIron, Syapse, Coda, IDT, GenScript |

Supplementary Table 4. Participant Methods for sharing data

| **Methods for Sharing Data** |
| --- |
| - Presentations, slide decks with visualizations and data tables |
| - Data Visualization Platforms such as PowerBI and Tableau |
| - Markdown notebooks - RMarkdown for R - Jupyter for Python |
| - Shared drives, email, Slack, USB drives |
| - HIPAA-compliant Box storage |
| - Mirth HL7 Engine |
| - Cloud-based platforms (e.g. AWS Deequ) |

Supplementary Table 5. Participant Methods for handling data access

| **Methods for Handling Data Access** |
| --- |
| - Internal IT departments - Linux chmod |
| - Role-based permissioning - Snowflake, AWS |
| - Third-party data management vendors |
| - Institution- or consortium-wide data governance councils |
